# Cyclic nucleotide signalling in malaria parasites

**DOI:** 10.1098/rsob.170213

**Published:** 2017-12-20

**Authors:** David A. Baker, Laura G. Drought, Christian Flueck, Stephanie D. Nofal, Avnish Patel, Maria Penzo, Eloise M. Walker

**Affiliations:** 1 Faculty of Infectious and Tropical Diseases, London School of Hygiene & Tropical Medicine, Keppel Street, London WC1E 7HT, UK; 2 Tres Cantos Medicines Development Campus, Diseases of the Developing World, GlaxoSmithKline, Severo Ochoa 2, Tres Cantos, 28760, Madrid, Spain

**Keywords:** malaria parasites, anopheles, cyclic nucleotides, phosphodiesterase, cyclase, *Plasmodium*

## Abstract

The cyclic nucleotides 3′, 5′-cyclic adenosine monophosphate (cAMP) and 3′, 5′-cyclic guanosine monophosphate (cGMP) are intracellular messengers found in most animal cell types. They usually mediate an extracellular stimulus to drive a change in cell function through activation of their respective cyclic nucleotide-dependent protein kinases, PKA and PKG. The enzymatic components of the malaria parasite cyclic nucleotide signalling pathways have been identified, and the genetic and biochemical studies of these enzymes carried out to date are reviewed herein. What has become very clear is that cyclic nucleotides play vital roles in controlling every stage of the complex malaria parasite life cycle. Our understanding of the involvement of cyclic nucleotide signalling in orchestrating the complex biology of malaria parasites is still in its infancy, but the recent advances in our genetic tools and the increasing interest in signalling will deliver more rapid progress in the coming years.

## 1. Introduction

Malaria parasites are fascinating organisms to study; research efforts, however, are primarily motivated by their devastating impact on humanity each year. Although the numbers of deaths and clinical cases have fallen dramatically over the last 15 years, mainly due to successful mosquito control programmes and effective drug treatments, there are still around 200 million cases of malaria resulting in over 400 000 deaths each year. Around 70% of these deaths are in children under 5 years old in Africa [1] infected with *Plasmodium falciparum* who have not built up sufficient natural immunity. Worryingly, the spread of insecticide-resistant mosquito vectors and drug-resistant parasites threatens to reverse the promising recent trend. The rate at which these organisms develop resistance vastly exceeds our ability to develop and bring new interventions to the field. There are a small number of very promising new antimalarial drugs that are undergoing clinical testing, some of which have been identified by phenotypic screening of compound libraries [2]. However, the attrition rate in drug discovery is very high and so the pipeline needs to remain productive. Trends in Pharma drug discovery seem to be shifting back towards target-based approaches, which emphasizes the importance of understanding the biology of malaria parasites at the molecular level and the identification of essential biochemical pathways that can be targeted with novel, small molecule inhibitors.

**Figure 1.**
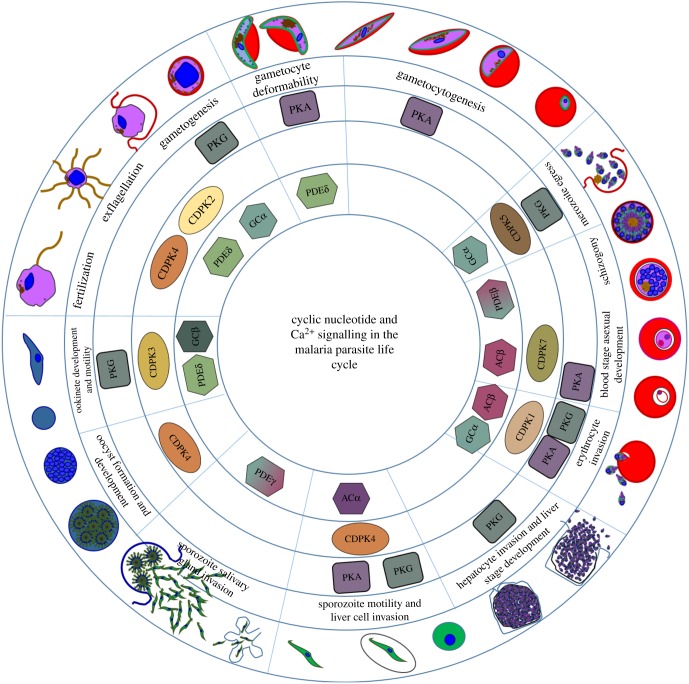
Stage-specific involvement of cyclic nucleotide signalling components and calcium-dependent protein kinases. A schematic depicting all the key stages of the complex *P. falciparum* life cycle. The inner circles depict which life-cycle stages have been associated with specific cyclic nucleotide signalling components and calcium-dependent protein kinases.

The malaria parasite life cycle is complex, with extended periods in the human host and also in the *Anopheles* mosquito vector which transmits disease between people (figure 1). When an infected female mosquito bites a person, sporozoites are injected into the bloodstream which travel to the liver where they invade hepatocytes. Here they divide asexually and eventually the hepatocyte ruptures to liberate thousands of merozoites from each infected cell. These merozoites must then invade red blood cells where they again replicate asexually by a process called schizogony whereby multiple daughter cells develop within a vacuole inside the infected erythrocyte. This blood stage cycle is responsible for all the pathology associated with malaria and, in the case of *P. falciparum,* takes 48 h. The synchronous rupture of mature schizonts to release the invasive merozoites causes the characteristic regularly spaced fevers associated with this infection. A subpopulation of the asexual blood stage parasites develops into male and female sexual precursors, called gametocytes, which mature in around 10 days. To continue the life cycle, gametocytes must then be taken up by a feeding mosquito where, following activation, they round up and gametes emerge from their host red blood cells. Male gametogenesis involves a visually and metabolically spectacular process known as exflagellation. Here, eight highly motile, flagellated gametes are generated from a single haploid male gametocyte within around 10 min of activation, allowing them to fertilize female gametes in the insect midgut. The resulting zygote develops into a motile ookinete which burrows through the mosquito midgut epithelium to form an oocyst. Asexual replication produces thousands of sporozoites, which when mature are released from the oocyst and migrate to the mosquito salivary glands to continue the cycle. It is clear that this complex life cycle must be tightly regulated to allow appropriate development and survival of all these highly specialized parasite forms in the varied environments they encounter. This has led researchers to investigate the signalling pathways that might be responsible for coordinating the timely progression of this intricate life cycle. This review will focus on current knowledge of the role of cyclic nucleotide signalling in regulating malaria parasite development.

**Figure 2.**
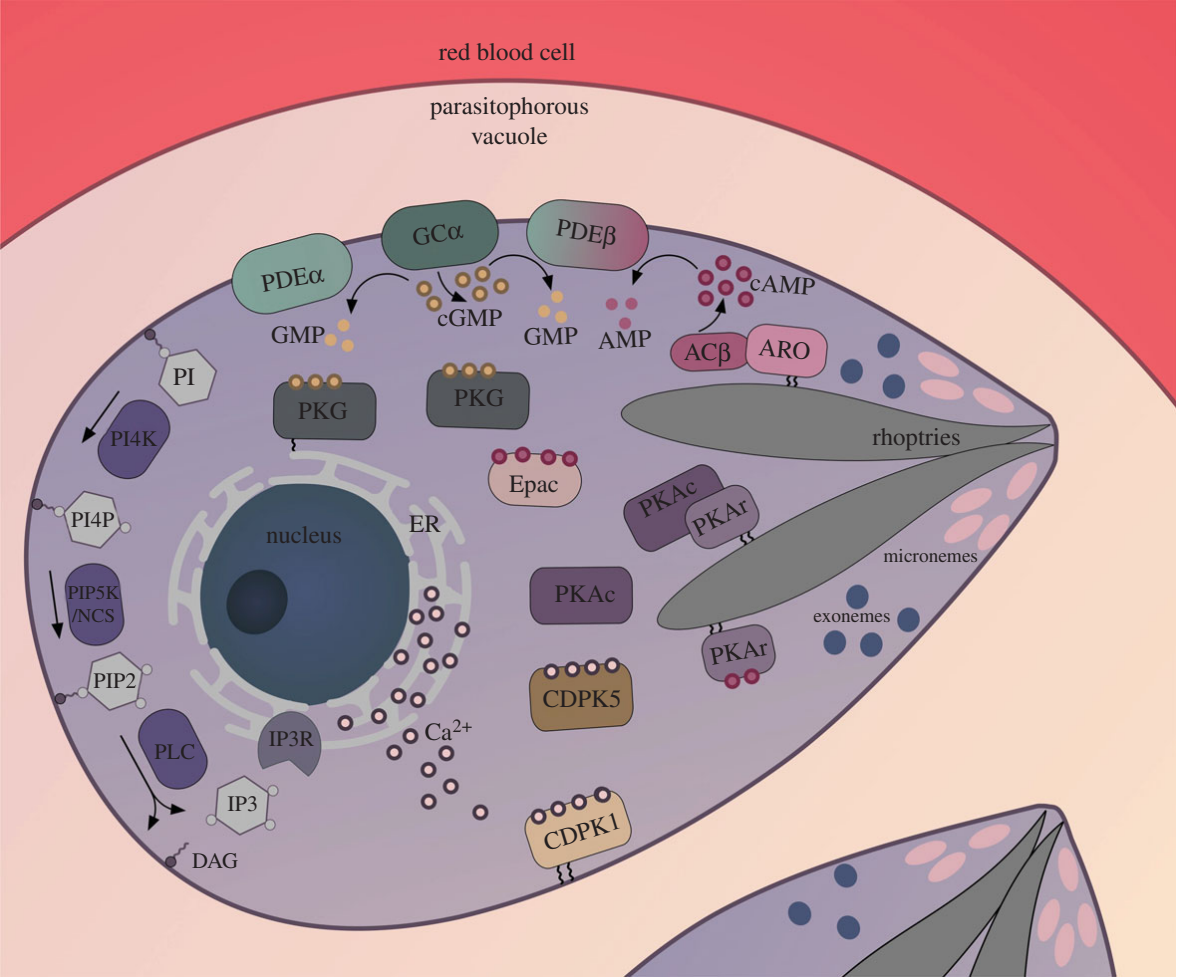
Localization of the key *Plasmodium* cyclic nucleotide signalling components in a merozoite prior to egress. A schematic showing the key cyclic nucleotide signalling players in a merozoite within a *P. falciparum* blood stage schizont. Some of the depicted cellular locations of pathway components are speculative (such as PDEα, PDEβ and GCα). Evidence suggests that a subpopulation of PKG is localized in the ER membrane, but the means of attachment is unknown. A rhoptry localization is depicted for ACβ based on data obtained from *Toxoplasma gondii*, where the location of the ACβ orthologue in the rhoptries is known to be dependent on the presence of ARO [7]. PKA is depicted as being rhoptry localized based on *P. falciparum* epitope tagging studies [8]. Also speculative is the existence of an (as yet) unidentified IP_3_-gated calcium channel (IP3R) for which there is pharmacological evidence.

Cyclic nucleotides are intracellular messenger molecules. Cyclic AMP (cAMP) and cyclic GMP (cGMP) are synthesized by adenylyl and guanylyl cyclase from ATP and GTP, respectively. Cyclic nucleotide signalling plays diverse functions across the phylogenetic tree. In mammals, for example, cAMP has multiple roles ranging from auditory function to mediating hormone action. cGMP is a central player in processes such as cardiac function and light detection in the eye. In single-celled eukaryotes, again cyclic nucleotides have myriad roles ranging from differentiation in *Dictyostelium* [3] to motility in ciliates [4]. cAMP and cGMP are hydrolysed by cyclic nucleotide phosphodiesterases (PDEs), which inactivate the messenger molecules. When cellular cyclic nucleotide concentrations reach threshold levels, following activation of a cyclase/inactivation of a PDE, they stimulate cAMP-dependent protein kinase (PKA) or cGMP-dependent protein kinase (PKG). The only other apparent cyclic nucleotide effector in malaria parasites is a molecule with putative cAMP-binding sites (PF3D7_1417400), designated PfEpac [5,6]. Prior to the availability of the full *P. falciparum* genome sequence, identification of some of these key enzymatic components in malaria parasites was a laborious process, but bioinformatic approaches have facilitated the task. Subsequent steps involved demonstration of biochemical activity and also functional analysis, which is still under way using genetic approaches (figure 2 and table 1). Although genetic manipulation of *P. falciparum* remains a significant challenge, the tools available have improved greatly in recent years and so advances in our understanding of these parasite signalling pathways will accelerate in the coming years. Functional studies of the key enzymatic components also facilitate our interpretation of pharmacological studies and in some cases necessitate a re-evaluation of the data.

**Table 1.** Phenotypes of mutants corresponding to pathway components. Details of the mutants that have been generated to date for the key players in malaria parasite cyclic nucleotide signalling (n.a., not applicable; n.d., not determined).

protein description	type of mutant	blood stage phenotype	sexual stage phenotype	sporozoite/liver stage phenotype	references
**adenylyl cyclase α**					
*P. falciparum* (PF3D7_1404600)	no mutant	n.a.	n.a.	n.a.	
*P. berghei* (PBANKA_1037500)	gene deletion	none	none	dramatically reduced apical exocytosis in response to liver cell transit	[9]
**adenylyl cyclase β**					
*P. falciparum* (PF3D7_0802600)	no mutant	n.a.	n.a.	n.a.	
*P. berghei* (PBANKA_1227600)	gene deletion	essential *	n.a.	n.a.	[10]
**guanylyl cyclase α**					
*P. falciparum* (PF3D7_1138400)	no mutant	n.a.	n.a.	n.a.	
*P. berghei* (PBANKA_0910300)	gene deletion	slow *			[10,11]
**guanylyl cyclase β**					
*P. falciparum* (PF3D7_1360500)	gene deletion	none	none	n.d.	[12]
*P. berghei* (PBANKA_1136700)	gene deletion	slow *	ookinete motility and mosquito midgut invasion defect		[6,11,13]
**phosphodiesterase α**					
*P. falciparum* (PF3D7_1209500)	gene deletion	none	n.d.	n.d.	[14]
*P. berghei* (PBANKA_1019350)	gene deletion	none			[10]
**phosphodiesterase β**					
*P. falciparum* (PF3D7_1321500)	no mutant	n.a.	n.a.	n.a.	
*P. berghei* (PBANKA_1419800)	gene deletion	essential *			[10]
**phosphodiesterase γ**					
*P. falciparum* (PF3D7_1321600)	gene deletion	none	none	n.d.	[12]
*P. berghei* (PBANKA_1419900)	no mutant	n.a.	n.a.	n.a.	
*P. yoelii* (PY17X_1421600)	gene deletion	peak blood stage parasitaemia reduced by 50%	none	reduction in salivary gland sporozoites (55%) and substrate-dependent gliding defect	[15]
**phosphodiesterase δ**					
*P. falciparum* (PF3D7_1470500)	gene deletion	none	gamete emergence defect	n.a.	[12]
*P. berghei* (PBANKA_0835600)	gene deletion	none	ookinete motility and mosquito midgut invasion defect	n.a.	[6]
**cAMP-dependent protein kinase catalytic subunit**					
*P. falciparum* (PF3D7_0934800)	no mutant	n.a.	n.a.	n.a.	
*P. berghei* (PBANKA_0835600)	gene deletion	essential *			[10]
**cAMP-dependent protein kinase regulatory subunit**					
*P. falciparum* (PF3D7_1223100)	over-expression	growth rate reduced by 78%	reduced stiffness of immature gametocytes		[5,16]
*P. berghei* (PBANKA_1438000)	no mutant	n.a.	n.a.	n.a.	
**cGMP-dependent protein kinase**					
*P. falciparum* (PF3D7_1436600)	inhibitor-resistant mutant **	block in schizont rupture	block in gamete egress	n.d.	[17,18]
*P. falciparum* (PF3D7_1436600)	FKBP DD knockdown	block in schizont rupture	n.d.	n.d.	[19]
*P. berghei* (PBANKA_1008200)	inhibitor-resistant mutant **	block in schizont rupture	block in gamete egress, ookinete motility defect	n.d.	[20]
*P. berghei* (PBANKA_1008200)	conditional gene excision	n.a.	n.a.	block in late liver stage development	[21]
**Epac-like protein**					
*P. falciparum* (PF3D7_1417400)	truncation	none	n.a.	n.a.	[22]
*P. berghei* (PBANKA_1025300)	no mutant	n.a.	n.a.	n.a.	

*PlasmoGem phenotype

**in conjunction with specific kinase inhibitor

## 2. Cyclase enzymes are distinct from those of other organisms

It has been reported that the properties of adenylyl cyclase activity derived from *P. falciparum* and the infected human red blood cell are distinct; for example, the host enzyme is activated by forskolin, whereas that of the blood stage parasite is not [23]. Malaria parasites have two adenylyl cyclases and two guanylyl cyclases that are topologically different to those of other organisms outside of the alveolate superphylum [24]. Although it is a predicted integral membrane protein, adenylyl cyclase alpha (ACα) is quite distinct from the membrane-associated mammalian G protein-dependent adenylyl cyclases. It has an apparently bifunctional structure with a C-terminal catalytic domain containing all the canonical residues required for cAMP synthesis and six predicted transmembrane domains, which have significant sequence identity to voltage-gated K^+^ channels. Expression in *Dictyostelium* and *Xenopus* confirmed the predicted adenylyl cyclase activity [25], while activity of the predicted voltage-gated K^+^ channel has so far not been confirmed [26]. However, in the related ciliate *Paramecium*, which has a gene with the same configuration, it was demonstrated prior to gene sequencing that a purified native adenylyl cyclase has the properties of a voltage-gated K^+^ channel [27]. It will be important to understand the physiological significance of a malaria parasite adenylyl cyclase enzyme which might transport K^+^ ions to regulate cAMP synthesis.

ACα (PBANKA_1037500) has been deleted in the *P. berghei* rodent malaria parasite [9]. Blood stage parasite growth and gametocyte production was not affected by gene deletion, nor was subsequent development in the mosquito, up to and including sporozoite growth and gliding. However, mutant sporozoites lacking this gene were unable to undergo apical regulated exocytosis in response to transit of sporozoites through hepatocytes (a prerequisite for infection to be established). Mutant sporozoites also showed a 50% reduction in invasion of a mouse hepatoma cell line [9]. The study also reported that pharmacological elevation of cAMP levels and inhibition of PKA activity, respectively, stimulated or reduced apical regulated exocytosis in both *P. yoelii* and *P. falciparum* sporozoites. Interestingly, in light of the predicted voltage-gated K^+^ channel domain of ACα, this study found that extracellular K^+^ is also required for sporozoite exocytosis. Together these results point to a role for cAMP signalling in apical regulated exocytosis in sporozoites. The involvement of intracellular messengers in albumin-stimulated sporozoite gliding was examined using pharmacological agents in a subsequent study. It was concluded that cAMP and PKA activity stimulated gliding and that elevation of cAMP levels could bypass the need for addition of albumin [28]. Using the PDE inhibitors IBMX and Zaprinast, the authors found that only the former stimulated sporozoite gliding and concluded that cAMP rather than cGMP signalling is involved in sporozoite motility. Curiously, it has previously been shown that IBMX does not inhibit PDE activity in *P. falciparum* blood stage parasites [16,29], but it is possible that it can inhibit PDE activity in sporozoites from rodent malaria parasites. The negative result obtained with Zaprinast (which is expected to raise cGMP levels) is seemingly at odds with the more recent observation that PKG activity is required for sporozoite gliding [30], but it is possible that the PDE activity in sporozoites is insensitive to Zaprinast. The authors next measured the effects of agents that influence mammalian heterotrimeric G proteins, which in mammals regulate transmembrane adenylyl cyclases (cholera toxin: stimulatory and mastoparan: inhibitory), to investigate whether this could be the mechanism that regulates cAMP synthesis during sporozoite gliding. Although the use of both of these agents suggested a role for heterotrimeric G proteins in sporozoite gliding, the apparent absence of the encoding genes in the parasite genome makes the results difficult to interpret. A more recent study investigated the effects of cholera toxin and mastoparan on *P. falciparum* sexual commitment. It was surprisingly found that mastoparan led to elevated cAMP levels and that cholera toxin had no effect, and it was concluded that the effects of both agents were unlikely to be via interaction with heterotrimeric G proteins [31].

The second adenylyl cyclase, ACβ, is an orthologue of the bicarbonate-sensitive soluble adenylyl cyclases [25]. So far, this gene has proved refractory to deletion in both *P. berghei* and *P. falciparum*, suggesting that it may be essential in the asexual blood stage. This is consistent with the fact that high levels of ACβ mRNA were measured in *P. falciparum* late blood stages [32]. Also, an antibody raised to *P. falciparum* PfACβ (PF3D7_0802600) reacted with both late schizonts and free merozoites [5]. Two distinct classes of soluble AC inhibitors (KH7 and 2CE) were found to kill *P. falciparum* blood stage parasites with LD_50_ values of 8.5 µM and 60 µM, respectively [33], further suggesting that PfACβ is essential in blood stages. Experiments using synchronized cultures showed that parasites died when KH7 was added during the first 24 h of the cycle, but were most sensitive between 24 and 31 h post-invasion. Expression of the N-terminal 785 amino acids of PfACβ (containing the predicted catalytic domains) in insect cells did not allow investigation of bicarbonate sensitivity of the enzyme, but it did reveal a distinct pH sensitivity (optimum 7.5), which led the authors to hypothesize that PfACβ acts as a pH sensor during blood stage development. Exposure of merozoites to a buffer containing low levels of K^+^, as would be encountered on entering the plasma post egress, triggered calcium release and discharge of AMA1 (PF3D7_1133400) from the micronemes. The buffer also triggered bicarbonate ion-sensitive cAMP synthesis that was reduced by addition of KH7 suggesting a key role for cAMP synthesis by PfACβ in these events [5,34]. It has been shown in *Toxoplasma* that ACβ is located on the outer face of the rhoptries facing the cytosol [7] and its rhoptry association is dependent on the presence of an interacting partner (AIP) of the armadillo repeats only protein (ARO). The latter is also known to be targeted to the rhoptries in *P. falciparum*, where it is attached to the cytosolic face of the rhoptry membrane by dual acylation [8]. It has also been observed that the *P. falciparum* PKA regulatory subunit has a similar location to PfARO (PF3D7_0414900) and is also probably targeted to the rhoptries [8]. Interestingly, PfARO is phosphorylated in a PKG-dependent manner at Ser_33_ and Ser_36_ [35], suggesting this may be a focal point for crosstalk between these two pathways during merozoite invasion.

The two malaria parasite guanylyl cyclases, GCα and GCβ, have unusual bifunctional structures [36], which are only shared by apicomplexans and ciliate relatives such as *Paramecium* and *Tetrahymena* [4]. They comprise a pair of C-terminal cyclase catalytic domains and two sets of six predicted transmembrane helices, reminiscent of the topology of mammalian G protein-dependent adenylyl cyclases. Key purine-binding residues in their catalytic domains, however, are clearly characteristic of guanylyl cyclases, and heterologous expression of the *P. falciparum* GCβ (PF3D7_1360500) catalytic domain [36] and the whole *Paramecium* enzyme in insect cells [4] have demonstrated that they are functional guanylyl cyclases. Curiously, amino acid motifs characteristic of the mammalian C1 AC catalytic domain are found in the C2 domain of the alveolate enzymes and vice versa. Evidence for membrane association was obtained using immunoelectron microscopy in both of these studies. The N-terminal region of all the alveolate GCs has sequence and structural homology with type IV P-type ATPases [4,11,36], but no functional activity has so far been demonstrated for this domain. Interestingly, proteolytic processing of the region linking the two functional domains of the *Paramecium* GC was observed in Sf9 cells, but whether this happens *in vivo* is not known. It will be intriguing to uncover the significance of the genetic linkage of these two distinct enzyme activities in apicomplexan parasites and their free-living relatives.

Early pharmacological studies [37–39] suggested cGMP as a possible regulator of exflagellation. Xanthurenic acid, a product of tryptophan catabolism and present in the mosquito midgut, is known to stimulate exflagellation [40,41]. Later it was shown that addition of xanthurenic acid to crude membrane preparations from *P. falciparum* gametocytes led to increased levels of cGMP, suggesting that xanthurenic acid may stimulate guanylyl cyclase activity (directly or indirectly) [42], again linking this pathway with exflagellation.

Transcriptomic studies have shown that the patterns of mRNA expression for PfGCα (PF3D7_1138400) and PfGCβ are very different (http://plasmodb.org), suggesting they each have roles in distinct life-cycle stages. Two studies have reported deletion of GCβ (PBANKA_1136700) in *P. berghei* which revealed a role in ookinete motility and mosquito midgut invasion [6,13]. Exflagellation occurred at a similar rate in mutant and wild-type lines, indicating that GCβ is not essential for this process in *P. berghei*. Similar findings were obtained following disruption of the *P. falciparum* GCβ in both the cyclase domain and the P-type ATPase domain. Gametogenesis was able to proceed normally despite the fact significantly reduced guanylyl cyclase activity was measured in mutant gametocyte membrane preparations [12]. It was deduced that the residual GC activity was due to the presence of GCα, which may also be expressed in gametocytes, and that this was sufficient for gametogenesis to occur normally. GCα has proved refractory to deletion in both *P. berghei* and *P. falciparum*, suggesting an essential role in blood stage development [6,11].

## 3. The parasite phosphodiesterases are all membrane-associated

There are four cyclic nucleotide phosphodiesterase enzymes (PDEα-δ) encoded in the malaria parasite genomes, but curiously there are potentially 18 in *Toxoplasma gondii* (TGGT1 strain; http://toxodb.org/toxo/). The malaria parasite PDEs are predicted to have up to six transmembrane domains, suggesting they are integral membrane proteins. The mammalian PDE superfamily is much more complex than that of *Plasmodium*. The mammalian PDEs are divided into 11 functionally divergent but structurally similar gene families, most containing several different genes. These genes are frequently spliced or transcriptionally processed to give over 100 PDE isoforms [43]. One family (PDE3) contains a single transmembrane domain, but, unlike *Plasmodium* PDEs, no mammalian PDE contains multiple membrane spanning domains. Mammalian PDEs contain 15 conserved residues, thirteen of which are conserved in all four PfPDE enzymes [44]. A number of transcriptional studies (available via http://plasmodb.org) have shown that the four *P. falciparum* PDEs exhibit life cycle stage-specific patterns of expression. PDEα (PF3D7_1209500) and PDEβ (PF3D7_1321500) are the predominant isoforms expressed in blood stage parasites and peak at the late trophozoite and schizont stages, while PDEγ (PF3D7_1321600) and PDEδ (PF3D7_1470500) are predominantly expressed in sporozoite and gametocyte stages, respectively.

A modelling study of the malaria parasite PDEs has been carried out using the human PDE9A crystal structure as a template [44]. Interestingly, the model could accommodate cGMP binding, but not cAMP binding, but as the authors say, roles for cAMP and PKA in the parasite have been established and so at least one of the four PfPDEs is likely to be able to hydrolyse cAMP and perhaps be dual specific. To date, it has been possible to measure catalytic activity for only PDEα (PfPDE1) by expressing the C-terminal catalytic domain in *Escherichia coli* [14,29]. This has shown that the enzyme specifically hydrolyses cGMP. The catalytic specificities of PDEγ and PDEδ for cGMP have been deduced from phenotype analysis of null mutants (but require confirmation; see below) while that of PDEβ remains unknown as it is the only one refractory to deletion. Expression of PDEα also allowed analysis of its sensitivity to some commonly used PDE inhibitors. Importantly, it was shown that both IBMX and theophylline, two PDE inhibitors that have been used in a number of studies on malaria parasites and have little or no activity against *Plasmodium* PDEs with EC_50_ > 100 µM [14,16,29]. It is important to note, in this respect, that some studies involving parasite PDEs use preparations in which the host red cell has not been removed and so human PDEs will be present, whereas some studies use saponin lysis to remove the host cell material. Yuasa and colleagues were able, however, to identify Zaprinast as a low micromolar inhibitor of recombinant PDEα and native PDE activity in *P. falciparum* blood stage parasites as well as blood stage growth [29]. Zaprinast has proved an extremely valuable tool (table 2) in a number of subsequent studies on cyclic nucleotides in apicomplexan parasites, and has helped provide proof of concept that malaria parasite PDEs are valid drug targets. The effects of analogues of the human PDE5 inhibitor, Tadalafil, on malaria parasites have also been reported, with EC_50_ values down to the sub-micromolar level [57]. Progress towards achieving selectivity for the parasite PDE by reducing activity against human PDE5 was also made by the same team by modifying the piperonyl group [58]. Another sub-micromolar inhibitor of malaria parasite PDEs, BIPPO, derived from a human PDE9 series, has an EC_50_ of 0.4 µM against *P. falciparum* proliferation and an IC_50_ of 150 nM against recombinant PDEα, but still retains an IC_50_ of 30 nM against human PDE9. Nonetheless, this compound will also be a very useful tool for studying cyclic nucleotide signalling in the parasite and could be an important lead for drug discovery [50].

**Table 2.** Pharmacological agents used to study cyclic nucleotide signalling in malaria parasites. Details of compounds that have been used to target *Plasmodium* proteins involved in cyclic nucleotide signalling. The parasite and host targets are indicated, if known, along with the potency reported for parasite or host cell and biochemical/phenotypic effects (n.a., not applicable; n.d., not determined).

compound	*P.falciparum* target	host target	published potency *P.falciparum* target	published potency host cell target	biochemical effect	published phenotypic effect
Compound 1	cGMP-dependent protein kinase	n/a	[IC_50_] 0.49 µM asexual blood stage growth [45]	no data avaliable	inhibition of parasite cGMP- dependent protein kinase	inhibtion of blood stage schizont rupture [17], inhibition of XA stimulated rounding up of gametocytes and gamete emergence [18]
Compound 2	cGMP-dependent protein kinase	n/a	[EC_50_] 395.0 nM ± 21.9 asexual blood stage growth [46]	no data avaliable	inhibition of parasite cGMP- dependent protein kinase	inhibtion of blood stage schizont rupture [17], inhibition of XA stimulated rounding up of gametocytes and gamete emergence [18]
Zaprinast	phospho-diesterases	phospho-diesterase 5	[IC_50_] 33.7 ± 1.3 µM for gametocytes and 3.0 ± 1.2 µM for schizonts [18]	[IC_50_] 1.3-3.2 µM PDE5 isoforms from cell lysate [47]	elevation of cellular cyclic nucleotide levels	induction of blood stage egress [48], induction of rounding up of gametocytes and gamete emergence [18]
Sildenafil	phospho-diesterases	phospho-diesterase 5 and 6	[IC_50_] 75.6 µM ± 1.6 for gametocytes and 22.5 µM ± 1.8 for schizonts [18]	[IC_50_] 3.5 nM PDE5 33 nM PDE6 [49]	elevation of cellular cyclic nucleotide levels	induction of rounding up of gametocytes and gamete emergence [18]
BIPPO	phospho-diesterases	n/a	[IC_50_] 0.4 µM ± 0.14 asexual blood stage growth [50]	no data avaliable	elevation of cellular cyclic nucleotide levels	induction of blood stage egress [50]
IBMX	n/a	phospho-diesterases	[IC_50_] >200 µM asexual blood stage growth [18]	[IC_50_] 5.8-26.2 µM [51]	elevation of host cell cyclic nucleotide levels?	n/a
H89	cAMP-dependent protein kinase catalytic domain	cAMP-dependent protein kinase catalytic domain	[IC_50_] 2.9 µM ± 1.6 asexual blood stage growth [52]	Ki 0.05 µM [53]	inhibition of cAMP- dependent protein kinase	inhibition of blood stage schizont development [52], inhibition of blood stage merozoite invasion [54], induces deformability in stage III gametocytes [16]
KT5720	cAMP-dependent protein kinase catalytic domain	cAMP-dependent protein kinase catalytic domain	[IC_90_] 10 µM asexual blood stage growth [54]	Ki 60 nM [53]	inhibition of cAMP- dependent protein kinase	inhibition of blood stage merozoite invasion [54], induces deformability in stage III gametocytes [16]
KH7	adenylyl cyclase β	soluble adenylate cyclase	[IC_50_] 8.5 µM asexual blood stage growth [33]	[IC_50_] 10 µM [55]	inhibition of cAMP synthesis	inhibiton of blood stage schizogony [33], inhibition of blood stage microneme release [5]
Forskolin	n/a	soluble adenylate cyclase	no effect on the blood stage enzyme [23]	[EC_50_] 2-10 µM [56]	stimulation of host cell cAMP synthesis	n/a

PDEα has been deleted in *P. falciparum*, showing that it is not essential for blood stage growth *in vitro*. Phenotype analysis suggested that ring-stage parasites appeared earlier in synchronized cultures of mutant parasites compared with wild-type, but it was not clear whether this was related to the absence of the PDEα gene. There was no evidence of upregulation of mRNA levels of the other three PDEs to compensate for the deleted gene. The study also highlighted the existence of two splice variants of PfPDEα (A and B), one of which encodes a protein with four predicted TM domains rather than six, but the significance of this is not known. cGMP-PDE activity in membrane fractions of the PDEα mutant was 20% lower than those from wild-type parasites, with no effect on cAMP-PDE activity. This is consistent with the findings of the recombinant expression studies that PDEα is cGMP specific. Interestingly, the authors also found that addition of excess cGMP to blood-stage membrane fractions from wild-type and PDEα knockout parasites greatly reduced cAMP hydrolytic activity in PDE assays, suggesting that one of the other PDEs expressed in blood stages is likely to be dual specific [14]. PDEβ, which has proved refractory to deletion in *P. falciparum* and *P. berghei* [10], is the most likely candidate for this activity, but this is yet to be demonstrated.

PDEδ has been deleted in both *P. falciparum* [12] and *P. berghei* [6], showing that this gene is dispensable for blood stage replication *in vitro*. Deletion of PDEδ in *P. falciparum* led to a 50% reduction in native cGMP hydrolytic activity in gametocytes compared with wild-type, with a corresponding twofold increase in cellular cGMP levels in late-stage gametocytes [12]. cAMP-PDE activity levels were consistently low, and indistinguishable between wild-type and mutant parasites. Interestingly, addition of Zaprinast to wild-type gametocyte membrane fractions reduced cGMP hydrolytic activity by around 75%, but it was totally ablated in PDEδ^−^ mutants. This suggests that the residual PDE activity is more sensitive to Zaprinast than PDEδ. Together these results indicate that PDEδ has cGMP hydrolytic activity and that at least one other cGMP-PDE is active in gametocytes. Although PDEδ mRNA and protein are present in gametocytes, their development up to maturity is not affected by the absence of this gene. However, upon stimulation of gametogenesis with xanthurenic acid, the ability of mutant parasites to round up was significantly reduced, and exflagellation of male gametocytes was reduced dramatically. Examination of mutant and wild-type parasites by immunofluorescence microscopy using an antibody against the human red cell membrane protein Band 3 showed that although most mutant parasites could partially round up following stimulation of gametogenesis, very few parasites had emerged from the encapsulating red cell. This suggests that elevated levels of cGMP, and perhaps premature PKG activation in gametocytes lacking PDEδ, have deleterious effects on gamete emergence. However, premature activation of gametocytes would be expected if this were the case, and so further work is needed to understand this phenotype. The results also indicate that PDEδ is important for regulation of cGMP levels during gametogenesis, and that both male and female gamete emergence is affected. This study also reported that PDEγ, which is also expressed in gametocytes at the mRNA level (PlasmoDB.org), was dispensable for *P. falciparum* blood-stage growth and gametocyte development *in vitro*, but in contrast to PDEδ^−^ mutants, there was no effect on gametogenesis [12].

Perhaps surprisingly, the phenotype was different in *P. berghei*; deletion of PDEδ had no effect on gamete emergence [6]. This suggests that another PDE might be able to compensate for its absence in *P. berghei* by maintaining cGMP concentrations in mature gametocytes below the level required for PKG activation. However, deletion of PbPDEδ had a dramatic effect on ookinete development. During the first 12 h of development, PDEδ^−^ parasites progressed to the expected banana-shaped ookinetes, but by 24 h they had ‘dedifferentiated’ into rounded or stumpy forms. This resulted in a more than 94% decrease in the number of oocysts that developed subsequently, compared with wild-type parasites. Interestingly, if these mutant ookinetes were injected into the haemocoel of the mosquito, transmission was restored to wild-type levels. Therefore, the mutant ookinetes were viable, but could not cross the epithelium of the mosquito midgut due to a lack of forward gliding movement. This abnormality was explained by some marked ultrastructural abnormalities (gaps in the inner membrane complex and misorientation of the underlying subpellicular microtubules) rather than a lack of motor activity [6]. Interestingly, the dedifferentiation phenotype could be reversed by addition of a specific PKG inhibitor, confirming that inappropriate activation of this enzyme had taken place following PDEδ deletion resulting in an elevation of cGMP levels. This conclusion was further strengthened by the fact that GCβ^−^/PDEδ^−^ double mutant ookinetes also had normal morphology. Another interesting finding was that a CDPK3^−^/PDEδ^−^double mutant glided normally (prior to dedifferentiation), thereby reversing the CDPK3^−^ mutant gliding phenotype. This indicated that elevated signalling through cGMP and PKG could compensate for the absence of this calcium-dependent kinase, which suggests there may be overlapping substrates for these two protein kinases.

Analysis of the localization of PDEγ using an epitope tagged *P. yoelii* transgenic line showed low-level expression in blood stages that partially co-localized with a marker of the endoplasmic reticulum (ER), but strong expression in sporozoites [15]. Although deletion of PDEγ caused a 50% reduction in peak blood-stage parasitaemia during the second half of a 20-day mouse infection, the gene is not essential for blood-stage replication. Furthermore, gametocyte and mosquito-stage development was unperturbed, including sporozoite formation in oocysts. However, there was a 55% reduction in the number of salivary gland sporozoites in mutant parasites compared with wild-type, and only very high sporozoite doses resulted in successful transmission to a proportion of mice, to establish a blood-stage infection. The authors demonstrated that this low level of transmission was due to the fact that the sporozoites lacking PDEγ are defective in substrate-dependent gliding motility. Transcriptome analysis of PDEγ^−^ mutant sporozoites showed upregulation of PDEβ and PDEδ and downregulation of several mRNAs encoding proteins involved in sporozoite motility and hepatocyte invasion [15]. Mutant sporozoites were found to have an almost 20-fold higher cGMP concentration than wild-type sporozoites, strongly suggesting this to be the cause of the defective gliding phenotype. Consistent with this assertion, the PDE inhibitor Zaprinast reduced sporozoite gliding in a dose-dependent manner. The authors point out that according to these and previous observations, it is possible that cGMP and cAMP may have opposing effects on sporozoite function and that crosstalk between the two pathways could occur [15].

## 4. PKG is essential in all of the key stages of the malaria parasite life cycle

**Figure 3.**
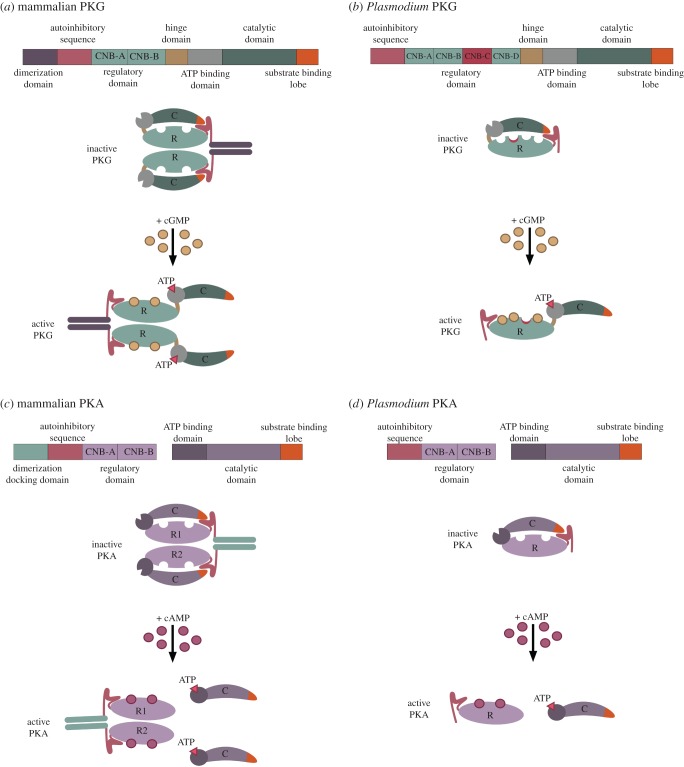
Cartoon of PKG and PKA domain organization and structure in mammals and *Plasmodium*. (*a*) Mammalian PKGs are homodimers encoded by single genes where dimerization is mediated by the dimerization domain. The inactive form of the enzyme adopts a conformation such that the autoinhibitory sequence interacts with the substrate-binding lobe, preventing access of substrates to the catalytic site. Binding of four cGMP molecules to the homodimer causes a conformational change, exposing the catalytic the domain to allow phosphorylation of substrates. (*b*) *Plasmodium* PKG is also encoded by a single gene; however, it lacks a dimerization domain and evidence suggests it forms a monomer. *Plasmodium* PKG contains four consensus cyclic nucleotide-binding domains (CNBs). One of these domains (CNB-C) is degenerate, therefore, the binding of only three cGMP molecules is required for activation of the enzyme. (*c*) Mammalian PKA is a heterotetramer consisting of two regulatory domains and two catalytic domains; however, unlike mammalian PKG, the regulatory and catalytic subunits are encoded by separate genes. The autoinhibitory sequence within the regulatory subunit binds to the substrate-binding lobe in the inactive form. The binding of four cAMP molecules results in the dissociation of the catalytic subunits from the regulatory subunits and activation of PKA. (*d*) *Plasmodium* PKA regulatory and catalytic subunits are also encoded by two separate genes; however, the regulatory subunit lacks a dimerization docking domain and the inactive enzyme is thought to form a heterodimer. The binding of two cAMP molecules results in dissociation of the subunits and allows binding of substrate proteins to the catalytic subunit.

The apicomplexan PKGs have both structural and biochemical properties that distinguish them from mammalian isoforms. For example, apicomplexan PKGs have four recognizable cGMP-binding domains (A, B, C, D); only three of these are functional and domain C is degenerate. By contrast, mammalian PKGs have only two cGMP-binding domains. In the absence of cGMP, PKG adopts a globular configuration where a pseudo-substrate, autoinhibitory sequence occupies the active site and so the enzyme is kept in an inactive state (figure 3). On binding cGMP, the conformation becomes more open and extended, and the autoinhibition is released, thereby exposing the active site to allow phosphorylation of substrate proteins. In *P. falciparum*, cGMP-binding site D has by far the highest affinity for cGMP and selectivity over cAMP. Crystallography carried out on this binding site has identified the key determinants of cGMP binding and identified a distinct capping mechanism. A conditional reverse genetic approach was taken to show that substitution of either of these capping residues is lethal to blood-stage parasites [59].

**Figure 4.**
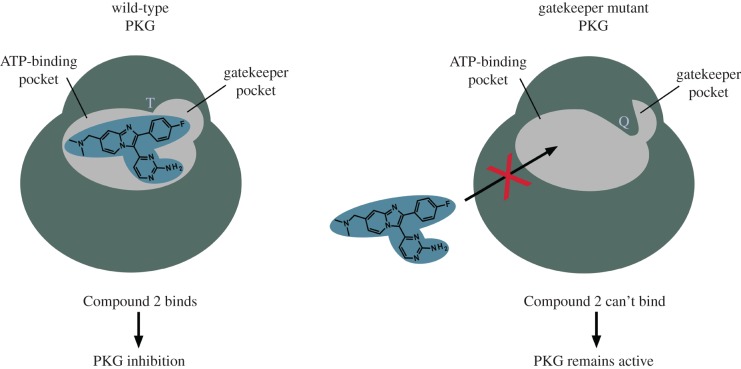
Mutation of the PKG gatekeeper residue confers inhibitor insensitivity. The ATP-binding pocket of PKG contains a small hydrophobic pocket. Inhibitors such as the imidazopyridine, Compound 2, compete with ATP for binding to PKG, with the fluorophenyl group of the inhibitor interacting with the hydrophobic pocket. Substitution of the threonine gatekeeper residue with a bulkier glutamine residue prevents binding of Compound 2 but the kinase remains functional with ATP still able to bind.

Two PKG isoforms have been identified in the coccidian parasites *Eimeria* and *Toxoplasma*. One of these is dually acylated at the N-terminus; modifications that allow membrane association [60]. Mammalian PKGII is membrane associated by myristoylation [61]. By contrast, in *Plasmodium* PKGs, the acylation motifs are absent and although a subpopulation of the *P. falciparum* PKG (PfPKG; PF3D7_1436600) apparently becomes associated with the ER membrane in late schizonts, the means of attachment is unknown [62]. Mutational analysis to examine the properties of the individual apicomplexan cGMP-binding domains has revealed that, unlike mammalian PKGs, they are relatively insensitive to 8-substituted cGMP analogues [63,64]. Progress with studying the malaria parasite PKG has been greatly facilitated by the existence of specific inhibitors which were generated through pioneering work by a group at Merck who identified a tri-substituted pyrrole (Compound 1) in a whole cell screen of *E. tenella*, a related apicomplexan parasite of chickens [65]. Using a range of biochemical approaches, they found that the target of Compound 1 is the *E. tenella* PKG and a subsequent medicinal chemistry effort led to the generation of a more potent imidazopyridine PKG inhibitor, Compound 2 [66]. At a similar time, another study carried out a biochemical analysis of the *P. falciparum* PKG and showed that it had a very similar structure and properties to the coccidian enzyme [64,67]. The two Merck PKG inhibitors were subsequently reported to be potent inhibitors of this malaria parasite PKG [18,45], and this proved crucial to functional studies when used in conjunction with an inhibitor-resistant transgenic line. This chemical genetic approach relies on the fact that the apicomplexan PKG has a structural feature, which is distinct from that of the human PKG and (importantly) the majority of serine threonine kinases, that allows inhibitor binding. The parasite PKG has a relatively small amino acid residue (threonine) in the so-called gatekeeper position (Thr_618_ in PfPKG). This is thought to allow access of the fluorophenyl group, shared by Compound 1 and Compound 2, to a small hydrophobic pocket which adjoins the kinase ATP-binding pocket. Substitution of the small gatekeeper residue with one with a bulkier side chain (such as glutamine or methionine) prevents access of the inhibitor to the gatekeeper pocket and renders the enzyme insensitive to it (figure 4). Introducing the gatekeeper mutant PKG into the parasite by complementation (*Toxoplasma* and *Eimeria* [68,69]) or allelic replacement (*P. falciparum* [17,18] and *P. berghei* [20]) confers inhibitor resistance to the transgenic line (the degree of resistance conferred varies with species and life-cycle stage). Comparison of the effects of the inhibitor on wild-type and gatekeeper mutant parasites first allowed demonstration that PKG is the primary target of the compounds, but importantly also allowed determination of the role of PKG in specific cellular processes in *Toxoplasma* [69] and *Plasmodium* [17,18,20,30]. In the presence of PKG inhibitor, wild-type *P. falciparum* gametocytes did not undergo rounding up and emergence from red cells when stimulated by xanthurenic acid or the PDE inhibitor Zaprinast (coupled with a reduction in temperature), whereas gatekeeper mutant parasites (expressing the PKG Thr_618_Gln substitution) could undergo rounding up normally, but exflagellation does not proceed, suggesting the involvement of a second, inhibitor-sensitive kinase in this process. This demonstrated a role for PKG in gametogenesis [18]. It is known that calcium signalling is essential for exflagellation [70], but interestingly, rounding up could occur normally in the presence of the calcium chelator BAPTA-AM, suggesting that PKG activity is upstream of calcium signalling and governs events up to and including emergence from the host cell. Using a chemical genetic approach, a recent study showed that CDPK4 is the major effector of calcium signalling downstream of PKG activation in male gametogenesis. It dissected multiple windows of kinase activity needed for DNA replication, mitotic spindle assembly and completion of cytokinesis to produce eight motile, flagellated male gametes. The same study also identified distinct CDPK4 substrates that translate each of the three bursts of CDPK4 activity into an appropriate cellular response [71]. Another recent study implicated an additional calcium-dependent protein kinase (CDPK2) in male gametogenesis. While female CDPK2 knockout gametocytes were able to emerge from the host cell normally, a proportion of flagellated male gametes were trapped inside the host red blood cell [72]. This suggests that these CDPKs might act in concert to regulate different aspects of gamete production.

Again using the gatekeeper mutant/inhibitor strategy, PKG activity was shown to be required for the rupture of blood stage schizonts [17]. Conditional knockdown of CDPK5 (PF3D7_1337800) caused a similar phenotype where schizonts could not rupture, but whereas PKG inhibition prevented proteolytic processing of key merozoite proteins by the essential protease PfSUB1 (PF3D7_0507500) [73], the CDPK5 phenotype was downstream of this proteolytic activity and cGMP signalling [74]. A subsequent study examined the underlying mechanism by which PKG inhibitors block blood stage schizont rupture and merozoite egress. It revealed that inhibition of PKG blocked release of PfSUB1 from the apical organelles known as exonemes, indicating that cGMP signalling and PKG activity are required for release of PfSUB1 from these organelles. Furthermore, in PKG inhibitor-treated schizonts, AMA1 could not be released from the micronemes onto the merozoite surface [48]. A conditional knockdown approach using a fusion with the destabilization domain (DD) showed that depletion of PKG led to a block in *P. falciparum* blood stage egress [19], a phenotype that is consistent with that obtained with the chemical genetic approach.

Another vital cellular process that was shown to be PKG-dependent using the gatekeeper mutant approach is calcium release from internal stores (figure 2). Elevation of cGMP levels stimulated by zaprinast, which triggers merozoite egress, was found to stimulate calcium release. This was measured by loading schizonts with the calcium-sensitive fluorescent dye, Fluo-4 AM. Addition of PKG inhibitor blocked calcium release in a dose-dependent manner. Analysis of phospholipids by mass spectrometry in *P. falciparum* wild-type and gatekeeper mutant schizonts treated simultaneously with zaprinast and Compound 2 showed PKG-dependent changes in phosphoinositide levels. For example, levels of PIP_2_ (which in higher eukaryotes would be hydrolysed by phospholipase C (PLC) to generate IP_3,_ which would in turn lead to activation of IP_3_-stimulated calcium channels leading to calcium release) were reduced by PKG inhibition. This study indicated that the process of PKG-dependent merozoite egress is mediated by calcium release which is linked to phosphoinositide metabolism [20]. To date it is not known what acts upstream of cGMP signalling to trigger egress, but there is evidence of events prior to PKG activation, with permeabilization of the parasitophorous vacuole membrane occurring 10–30 min before its PKG-dependent breakdown [75]. Interestingly, in *Toxoplasma* it has been shown that the plant hormone abscisic acid triggers egress in a calcium-dependent manner [76], but it remains to be seen whether this molecule has a role in malaria parasites.

The gatekeeper mutant and PKG inhibitor have also been used in conjunction with global phosphoproteomics to identify potential PKG substrates in *P. falciparum* schizonts. Peptides derived from schizont proteins from PKG inhibitor-treated and untreated cultures of wild-type and gatekeeper mutant parasites were labelled with isobaric tags and analysed using quantitative mass spectrometry. A total of 107 phosphosites from 69 individual proteins were regulated in a PKG-dependent manner, revealing involvement of cGMP signalling in a range of cellular processes including egress/invasion, motor function, signal transduction, ion and protein transport, and chromatin regulation. Interestingly, PKG-dependent phosphosites were identified on GCα, PDEβ and ACβ suggesting feedback loops involving cGMP and possible crosstalk with cAMP signalling. Work is under way to determine the physiological significance of a number of these phosphosites in key proteins. This approach identified PKG-dependent phosphorylation events in schizonts, but in many cases it is likely that the proteins are actually phosphorylated by kinases that act downstream of PKG. One protein that was found to be a direct substrate of PKG in schizonts is CDPK1 (PF3D7_0217500), suggesting that PKG may play a dual regulatory role in calcium signalling, stimulating calcium release and direct phosphorylation of calcium-dependent kinases [35]. A recent study reported the successful generation of a *P. falciparum* CDPK1 gatekeeper mutant (harbouring a Thr_145_Met substitution). This line had a significantly reduced kinase activity, but interestingly had a greater sensitivity to the PKG inhibitor Compound 2 than wild-type parasites. This was interpreted as indicating that PKG was probably compensating for the reduced activity of the mutant CDPK1 enzyme. We believe it is more likely that the increased sensitivity of this line to Compound 2 is due to the fact that CDPK1 is also a target of this inhibitor and that because the gatekeeper mutant CDPK1 cannot bind Compound 2, the amount of free compound available to block PKG function is elevated, resulting in a lower EC_50_ value than in wild-type. The increased transcript levels of CDPK5 and CDPK6 (PF3D7_1122800) measured by the authors in the mutant line probably indicate that these closely related kinases compensate for the reduced CDPK1 activity [77]. CDPK7 has been shown to be important for early blood-stage development and is therefore likely to operate independently of PKG. This kinase is unique among the CDPKs in having a pleckstrin homology domain that has been shown to bind to PIP_2_ and is proposed to guide its subcellular localization [78].

PKG has also been shown to play an essential role in *P. berghei* ookinetes. The PKG inhibitor Compound 1 prevented ookinete gliding with an EC_50_ of 100 nM. PKG fused to the fluorescent reporter GFP indicated a largely cytosolic location in ookinetes [79]. A subsequent study examined in more detail how cGMP signalling and PKG activity might regulate ookinete gliding. The study used quantitative mass spectrometry to analyse cGMP-dependent phosphorylation events using two parallel genetic approaches. The first used a GCβ knockout line and the second used the PKG inhibitor Compound 2 in conjunction with a *P. berghei* gatekeeper mutant parasite line (expressing a Thr_619_Gln substitution). Changes in phosphorylation levels in either of the approaches detected key sites in PKG itself as well as in GCβ and PDEδ, showing potential mechanisms for self-regulation of the pathway. Interestingly, regulation of proteins involved in phosphoinositide metabolism and vesicle trafficking was measured in both of these two approaches in *P. berghei*. For example, phosphopeptides identified in three of the four PI4 K (PBANKA_110940; PBANKA_072200) and PIP5 K (PBANKA_020310) isoforms were reduced significantly, suggesting that phosphoinositide metabolism is required for ookinete motility and that it is regulated by PKG. The lack of any change in phosphosites on PLC suggests that regulation is at the level of sequential phosphorylation of phosphatidylinositol (PI) rather than at the level of PIP_2_ hydrolysis [20]. Interestingly, *P. berghei* ookinetes containing a PI4 K (PBANKA_110940) phosphomutant (Ser_534_Ala) showed a reduced gliding speed compared with wild-type parasites. Furthermore, a *P. berghei* line expressing HA-tagged PIP5 K (PBANKA_020310) showed a change in distribution from the cell periphery to the cytosol when treated with PKG inhibitor. Intriguingly, this PIP5 K has a predicted bifunctional structure with an N-terminal domain with homology to the EF-hand-containing neuronal calcium sensors, a subfamily of which activate guanylyl cyclase in mammals. The *P. falciparum* orthologue (PfPIP5 K/NCS; PF3D7_0110600) has been shown to be activated by ADP-ribosylation factor (ARF1) to generate PIP_2_ [80], providing another potential link between phosphoinositide metabolism and vesicular trafficking. Both of these observations strongly suggest a link between PKG and phosphoinositide metabolism. Analysis of ookinete phospholipids by quantitative mass spectrometry in the presence and absence of PKG inhibitor revealed reduced levels of PI4P and PIP_2_ synthesis following PKG inhibition. This in turn suggested a potential link to calcium release via hydrolysis of PIP_2_ (by PLC) to give IP_3_ (and diacylglycerol) that in other systems activates calcium channels. An IP_3_-gated calcium channel sequence has not been identified in *Plasmodium* genomes, but it is likely that a protein exists in the parasite which performs this role. A transgenic *P. berghei* line expressing a fluorescent calcium reporter showed clearly that inhibition of PKG led to rapidly reduced levels of cytosolic calcium in ookinetes. Furthermore, the PKG-mediated stimulation of gametogenesis by reduced temperature and xanthurenic acid was also shown to stimulate calcium release, and this was detectable just 10 s after stimulation [20]. The *P. berghei* line expressing gatekeeper mutant PKG was used to demonstrate that the effects of PKG inhibition were specific.

In addition to its role in blood-stage egress and invasion, gametogenesis and ookinete motility, PKG has also been shown to be essential for late-liver-stage development in *P. berghei*. As it was not possible to delete the gene, an elegant conditional knockout approach was used, whereby gene excision was triggered during mosquito stage development by a drop in temperature [21]. Gene excision was detectable in the sporozoite stage. Injection of mice with these sporozoites did not lead to a subsequent blood-stage infection. Examination of liver stages in HepG2 cell cultures showed that development could apparently proceed normally up to 40 h post-infection; however, after 72 h, cultures appeared blocked at the late liver schizont stage with few merosomes (vesicles containing merozoites) released. A follow-up study using the PKG inhibitor Compound 1 showed that it blocked sporozoite gliding, invasion and infection, but in the light of the previous findings this was interpreted as off-target activity. However, a more recent study used the gatekeeper mutant approach in *P. berghei* for both PKG and CDPK4 (PF3D7_0717500) in conjunction with either a PKG inhibitor or a bumped kinase inhibitor (BKI 1294), used as a CDPK4 inhibitor, to demonstrate that PKG is in fact required for sporozoite gliding motility (and that Compound 1 was on target), microneme exocytosis and hepatocyte invasion, with CDPK4 also having a role in hepatocyte invasion [30]. The findings of this paper are seemingly at odds with the earlier work described above, which suggested that PKG is not required for sporozoite function. The authors pointed out that this discrepancy is likely to be due to residual PKG protein synthesized prior to conditional excision carried over into the sporozoites [30].

It is clear from the studies described above that PKG plays an essential role at almost every stage of the malaria parasite life cycle (figure 1). This emphasizes that this molecule might be worth pursuing as an antimalarial drug target. *In vivo* proof of concept has been established following a medicinal chemistry effort to optimize the imidazopyridines against the *P. falciparum* PKG. Oral dosing of a *P. falciparum* mouse model was able to clear blood-stage infection as well as block transmission of gametocytes to mosquitoes. This study also reported co-crystal structures that confirmed the importance of compound interactions within the gatekeeper pocket of the kinase [46]. An important related finding that has emerged over time is that PKG usually acts in concert with a stage-specific CDPK (figure 1). In some cases (e.g. gametogenesis and blood stage merozoite egress), it appears that PKG triggers release of calcium into the cytosol, which leads to the activation of the corresponding CDPK, but evidence suggests that PKG can also phosphorylate the partner CDPKs, which might fine-tune the stage-specific calcium response.

## 5. A role for PKA in multiple life-cycle stages is emerging

Unlike PKG, the cAMP-dependent protein kinase, PKA, is made up of distinct polypeptides encoded by separate genes (figure 3). The inactive enzyme is made up of a catalytic subunit (PKAc) and a regulatory subunit (PKAr) which contains a pair of cAMP-binding sites. When cAMP binds to PKAr, the complex dissociates and PKAc is thereby activated and can bind to substrate proteins which are then phosphorylated. When bound to PKAr, the PKAc enzyme is inactive, with part of PKAr binding to the active site via a substrate-like autoinhibitory sequence. In mammals, there are four regulatory subunits (two type R1 and two type R2), and a complex of four polypeptides made up of two catalytic subunits (three C types) and two regulatory subunits constitutes the inactive heterotetrameric state [81]. By contrast, in *Plasmodium*, there are single genes encoding both PKAc (PF3D7_0934800) [82] and PKAr (PF3D7_1223100) subunits. A unique feature of the malaria parasite PKA regulatory subunit sequence is that the dimerization domain characteristic of PKAr in other species is absent [83]. The sequence of *Plasmodium* PKAr suggests it may be a hybrid of the human R1 and R2 types [84], and crystallographic analysis confirmed that it has features that are characteristic of both human R1 and R2 types [85].

In mammals, the localization and function of PKA is determined by interaction with a family of A kinase activating proteins (AKAPs) [86]. AKAPs ensure that PKA activity is targeted to the correct cellular compartment and ensures its specificity of response to discrete cellular stimuli. However, the dimerization/docking domain of PKAr that in other species allows tethering to AKAP is absent in the *Plasmodium* PKAr sequence [83]. To date only one gene encoding an AKAP-like protein (PfAKAL; PF3D7_0512900) has been identified in *P. falciparum*, and while no direct interaction with the parasite PKAr could be detected, there was evidence that it binds to Pf14–3-3 (PF3D7_0818200; which in turn binds to PKAr) and also to AMP and cAMP [87], suggesting a divergent AKAP mechanism in *Plasmodium* species. Intriguingly, *P. falciparum* Rab5A (PF3D7_0211200) and Rab7 (PF3D7_0903200) proteins have also been identified as potential PKAc interactors, and the authors suggested that Rabs may act as surrogate AKAPs [88,89].

The reported role of PKA in *P. falciparum* is varied, acting at multiple stages of the parasite life cycle. An early study detected *P. falciparum* blood stage parasite proteins that were phosphorylated in a cAMP-dependent manner, and further isolated a kinase activity that was cAMP-dependent thereby revealing the presence of PKA in malaria parasites [90]. The *P. falciparum* PKA activity detected in asexual blood-stage parasites was shown, as with the human red-cell-derived activity, to be sensitive to the PKA inhibitors H89 and PKI [52]. However, a later study using expression of the *P. falciparum* PKAc in a cell-free system demonstrated that it was much less susceptible to these inhibitors than mammalian PKA [91,92]. Two *P. falciparum* cloned lines (LE5 and T9/96) that differ in their ability to produce gametocytes were examined to determine whether they have differences in their adenylyl cyclase or PKA activity. This was prompted by a report that addition of 1 mM cAMP to static cultures of the *P. falciparum* Z isolate could trigger conversion of almost 100% rings to gametocytes [93]. While AC activity was the same in both parasite clones, PKA activity was found to be significantly higher in the gametocyte-producer line, suggesting that this could reflect a causal relationship [90]. By contrast, a subsequent study attempting to reproduce the experiment performed by Kaushal *et al.* [93] reported a negative effect of cAMP on asexual growth and gametocytogenesis [94], potentially indicating a cryptic pleiotropic effect of this molecule on the parasite cell cycle. A number of groups have investigated this enigmatic link between cAMP signalling and gametocyte formation. For example, Dyer & Day showed that treatment of malaria parasites with cholera toxin (which in mammals stimulates AC by activation of the G_s_α class of heterotrimeric G proteins) enhanced gametocyte formation [95]. The absence of genes encoding heterotrimeric G proteins from the *Plasmodium* genome, however, makes these results difficult to interpret. Others have found that cholera toxin has an effect on malaria parasites, but concluded that its target is unclear [31].

It has been reported that the human hormone melatonin causes *in vivo* synchronization of the rodent malaria parasite *P. chabaudi* and that this is achieved through calcium signalling [96]. Intriguingly, the same group has shown that 100 nM melatonin can promote early maturation of schizonts and stimulate elevated cAMP levels and PKA activity in *P. falciparum* blood stages in a phospholipase C-dependent manner, suggesting the involvement of calcium signalling [97]. The same study also showed that addition of 20 µM 6-Bz-cAMP, a cell-permeable cAMP analogue, can induce Ca^2+^ release in the parasite cytosol, implicating cAMP in modulating downstream calcium-dependent processes. No change in cAMP was stimulated by forskolin, suggesting that the human red cell AC was absent from the preparation. The same two pathways have been implicated in mediating the effects of melatonin in *P. chabaudi* [98]. In a more recent study, this group have examined gene expression changes caused by melatonin and cAMP using RNA-Seq. Addition of melatonin led to a change in expression of 38 genes in trophozoites (101 with cAMP) which was dependent on the presence of an intact *P. falciparum* protein kinase 7 (PK7; PF3D7_0213400) gene; *P. falciparum* PK7 knockout line is unable to respond to melatonin. Gene ontology and KEGG pathway analysis indicated that components of the ubiquitin–proteasome system (UPS) pathway were regulated by both treatments in trophozoites [99].

It is known that the parasite transports many proteins into the host red cell; some of these are involved in the binding of infected red cells to host endothelial cells and some are involved in nutrient uptake and disposal. It has been shown in uninfected red cells that anion conductance can be activated by cAMP [100] and that PKA plays a role in anion conductance in the membrane of *P. falciparum*-infected red blood cells [83]. Injection of recombinant *P. falciparum* PKAr into the infected red cell using a patch-clamping pipette led to a decrease in anion conductance. Whether this is due to inhibition of erythrocyte-derived PKA is unclear, as it may be that PKAr injected into the erythrocyte cytosol cannot access parasite PKA contained within internal limiting membranes. Furthermore, transgenic overexpression of PfPKAr had an inhibitory growth effect on the parasite, in conjunction with decreased anion conductance, indicating a role for this pathway in parasite cell cycle progression. This growth defect could be reversed by addition of IBMX (a PDE inhibitor expected to elevate cAMP levels) to the culture medium, which may indicate a role of erythrocyte host derived PDEs in this process as IBMX does not inhibit parasite PDEs (table 2). What is not clear is whether the cAMP production and transduction machinery involved in anion conductance in parasite-infected red cells is derived from the red cell or the parasite [100,101]. A recent conditional knockout study has clearly implicated the parasite derived RhopH complex in facilitating the increase in anion conductance [102].

A role for PKA in *P. falciparum* red cell invasion has also been reported [54]. AMA1, a protein released from the micronemes onto the merozoite surface and involved in tight junction formation, was shown to be phosphorylated at Ser_610_ in the cytoplasmic tail. Pharmacological and genetic evidence indicated that this event was carried out by the parasite PKA. The event was enhanced by addition of a cAMP analogue and reduced by PKA inhibition in parasite lysates. Substitution of AMA1 Ser_610_ with an alanine using a conditional transgenic approach led to a dramatic reduction in merozoite invasion [54]. A later report confirmed the PKA-dependent phosphorylation of AMA Ser_610_, and showed that phosphorylation of Ser_610_ is an initial step that is needed before subsequent phosphorylation of Thr_613_ by the parasite glycogen synthase kinase 3 (GSK3; PF3D7_0312400) required for efficient invasion [103]. As mentioned above, a regulatory role for cAMP and PfACβ in calcium release and microneme secretion has been demonstrated using a low K^+^ buffer system [5]. Free K^+^ ions cannot readily cross membrane barriers and require specific ion channels to facilitate this process. In the light of this, the details of how this drop in K^+^ concentration is sensed by the parasite and how it relates to a change in cAMP within the cell remain enigmatic. Evidence was presented suggesting that, once elevated, cAMP acts through two different pathways. First, experiments using *in vitro* kinase assays and a transgenic line overexpressing PfPKAr pointed to a role for PKA activity in regulating microneme release. Second, evidence derived from a pharmacological approach implicated an exchange protein directly activated by cAMP (Epac)-like molecule (PfEpac; PF3D7_1417400) in the calcium mobilization required for microneme release and merozoite invasion [5]. PfEpac contains consensus cyclic nucleotide-binding domains [6], but lacks other characteristic Epac signatures. In other organisms, Epac stimulates a cAMP-dependent calcium release by activating PLC via the small G protein rap1, but no orthologue has been identified to date in *P. falciparum*. Interestingly, it has been shown recently that culture-adapted *P. falciparum* isolates have a high propensity to undergo truncation of the PfEpac gene [22], suggesting that expression of the full-length gene represents a fitness cost *in vitro* and a non-essential role in blood-stage parasites.

A global phosphoproteome analysis carried out on *P. falciparum* schizonts revealed that 425 of the 2541 unique phosphosites conformed to one of the mammalian PKA substrate consensus sequences ([R/K] R/K × S/T), suggesting that this kinase is likely to have a key role at this stage of the life cycle [104]. Bioinformatic approaches determined that a number of these potential PKA substrates are found in the ‘locomotion and entry into host’ Gene Ontology category which is required for merozoite invasion. *In vitro* phosphorylation of a synthetic peptide derived from Myosin A and recombinant CDPK1 and GAP45 (glideosome-associated protein 45; PF3D7_1222700) by bovine PKA suggested that these motor proteins could be PKA substrates in *P. falciparum* schizonts. The analysis also revealed phosphorylation in schizonts of several proteins involved in phosphoinositide metabolism, suggesting a key role for phosphatidylinositol signalling at this stage of the life cycle [104]. The authors point out that four phosphatidylinositol kinases are phosphorylated at sites resembling consensus PKA sites; however, consensus PKA and PKG sites are very similar [105], and previous work on *P. berghei* ookinetes has suggested that phosphatidylinositol kinases are phosphorylated in a PKG-dependent manner [20]. The question of whether this phosphorylation is carried out directly by PKG or indirectly acting via downstream PKA phosphorylation will need further work.

A role for cAMP and PKA has also been found in gametocyte development. It is thought that the non-deformable nature of immature *P. falciparum* gametocytes allows them to sequester in bone marrow tissue to prevent splenic clearance [106–109]. However, to transmit to mosquitoes, mature gametocytes must re-enter the circulation and evidence suggests that to do this they need to become deformable. Using a microbead separation system designed to emulate splenic filtration, agents which promote cAMP signalling (such as cAMP analogues, AC activators and PDE inhibitors) through PKA were found to cause gametocytes to become more rigid (and retard their passage through the micro bead matrix), whereas reduced signalling stimulated by, for example, PKA inhibitors (but not a PKG inhibitor) caused the gametocytes to become more deformable [16]. In a parallel genetic approach in the same study, a line that overexpresses the regulatory subunit of PfPKA showed higher deformability in immature gametocytes. In addition, gametocytes from a PfPDEδ knockout line displayed a twofold increase in cAMP levels and a concomitant decrease in deformability of mature stages. Whether this effect on gametocyte cAMP levels is directly or indirectly attributed to PfPDEδ remains to be established, as deletion of PfPDEδ has been previously shown to reduce cGMP hydrolysis. Taken together these results suggest that cAMP signalling plays a role in the ability of gametocytes to sequester and re-enter the circulation on maturation [16]. A subsequent paper by this group showed that gametocyte rigidity is mediated in part by the interaction of the C-terminal tail of members of the STEVOR proteins (encoded by a sub-telomeric multigene family) with the red cell cytoskeleton ankyrin complex. Furthermore, it was shown by a pharmacological and genetic approach that this interaction requires phosphorylation of STEVOR (PF3D7_0631900) at Ser_324_ by PKA, a residue conserved across the STEVOR family [110]. Whether this is via host PKA or by PfPKA remains to be resolved, but due to the localization of STEVOR in the erythrocyte cytosolic membrane it may be the former.

Finally, a note of caution. Several of the studies described above, including our own, have relied to some extent on the use of pharmacological agents (table 2). In many cases, these reagents have been well characterized in other systems such as mammalian cells, but far less is known about their targets in malaria parasites. Where possible, such agents should only be used in parallel with genetic approaches to give additional confidence of on target activity. We are very fortunate to have potent apicomplexan PKG inhibitors that can be used in conjunction with inhibitor-resistant transgenic lines to determine whether or not the inhibitor is on target during a particular life-cycle event. For example, it has been shown using this approach that the pyrrole, Compound 1, primarily targets PKG during events such as rounding up of *P. falciparum* gametocytes, merozoite egress and sporozoite gliding/hepatocyte infection; however, the compound has alternative primary targets during exflagellation [18] and early-blood-stage development [17]. These observations are likely to be more widely applicable, suggesting that many pharmacological agents might be reliable at some life-cycle stages but not at others.

## 6. Conclusion

In recent years, important steps have been made in determining the function of cyclic nucleotide signalling in malaria parasites, but recent advances in our genetic tools will ensure that we will have a more complete picture in the next 5–10 years. One of the big remaining questions is what operates upstream of cyclic nucleotide signalling in malaria parasites; only in gametogenesis do we have an answer to this. A comprehensive research effort will be required to determine the biological significance of the individual phosphorylation events driven by PKA and PKG. It will also be fascinating to discover how these two pathways interact to coordinate merozoite egress and invasion. Another important question remains regarding how specificity is maintained when PKA and PKG have such similar consensus substrate sequences. How is it that all proteins having this basic amino acid motif are not haphazardly phosphorylated by either kinase because they are both expressed in late schizonts and merozoites? However, we do not know whether peaks of cAMP and cGMP coincide temporally within schizonts and free merozoites. It is also likely that, when active, the two kinases are located in distinct cellular compartments and/or are tethered within distinct protein complexes that bring them into the proximity of their respective substrates. The revelation that enzymatic components of the pathways play such pivotal roles in life-cycle progression has provided important target validation data for drug discovery projects. In partnership with medicinal chemists, it is possible that effective future antimalarial drugs will be developed that selectively target cAMP and cGMP signalling in malaria parasites.

## Data accessibility

This article has no additional data.
